# *“Well*,* these are deeply rooted customs”*: a qualitative study on nutritional practices among postpartum mothers in Oaxaca, Mexico

**DOI:** 10.1186/s40795-026-01414-0

**Published:** 2026-06-30

**Authors:** Marian Marian, Ramona L. Pérez

**Affiliations:** 1 University of California San Diego, 9500 Gilman Drive, La Jolla, San Diego, CA 92093 USA; 2 Department of Anthropology, San Diego State University, San Diego, CA USA

**Keywords:** Postpartum period, Nutrition practices, Breastfeeding, Postnatal women, Mexico

## Abstract

**Background:**

Maternal nutrition during the postpartum period is a critical time for mothers as they recover from pregnancy and delivery and continue to support their infant through breastfeeding. As Mexico goes through a nutrition transition away from traditional, home-prepared meals to more processed foods, it is unknown what the current dietary choices of women are after pregnancy.

**Objective:**

This study aimed to explore the nutritional practices of women in Southern Mexico during the postpartum period.

**Methods:**

A qualitative study, guided by the Ecological Systems Theory and the Intersectionality Framework, was conducted involving 25 low-income women with children under the age of five in the cities of Oaxaca and Puerto Escondido, Oaxaca, Mexico. Data were collected through in-depth interviews between June and December 2023. Interviews were audio-recorded, transcribed verbatim in Spanish, coded, and analyzed using NVivo R1 following a grounded theory approach.

**Results:**

Three main themes emerged: (i) maternal diet after a C-section, (ii) food practices during the breastfeeding period, and (iii) nutrition supplements after the delivery. These findings reveal that compared to women who deliver vaginally and are not advised on a specific diet during the postnatal recovery, women who deliver via C-section usually have a selective diet, based on recommendations from health providers and family members. Their diet consists mainly of fruit, vegetables, and broths with a restriction from consuming seafood and pork. A common traditional hot beverage, *Atole*, was frequently consumed by postnatal women to increase their milk supply. Iron and folic acid were nutritional supplements mostly consumed during the postnatal period by women with a greater need to replenish nutrients lost during childbirth or the postnatal period.

**Conclusion:**

This study highlighted the significance of understanding the nutritional habits that postnatal women in Puerto Escondido and Oaxaca City practice, as they are driven by customs and focus on the recovery of the mother and the development of the baby. These findings could be used to inform culturally relevant policies. Still, as postnatal care continues to be understudied in Mexico, future research is needed to identify any gaps in the nutrition of the mothers during puerperium.

## Background

The puerperium, better known as the postpartum or postnatal period, is a six-week period immediately following childbirth that is critical for the health of both the mother and baby. Healthy eating for the mother is prioritized and recommended as the body recovers and returns to its pre-pregnant state as well as the physiological demands of breastfeeding [1, 2]. Micronutrient excess or deficiency during the postnatal period can affect the quality and quantity of breast milk as well as short- and long-term maternal and child health [3]. 

In Mexico, women currently face healthcare and nutritional challenges during the pregnancy and postpartum periods. While the levels of breastfeeding in this country have increased, its prevalence continues to be close to 35%, which is lower than the 50% established by the World Health Organization (WHO) and lower compared to other countries in the Latin American region [4, 5]. Micronutrient deficiency is very common among pregnant women in this country [6]. The national prevalence of anemia among Mexican pregnant women is 35%; however, these levels are greater among women living in rural areas (42%) and adolescent girls (46%) [7]. Nevertheless, according to the National Survey of Health and Nutrition, more than 80% of women receive adequate prenatal care, more than 70% get iron and vitamin supplementation, and more than 90% get folic acid supplementation [6]. It is important to state that there are no current statistics on the anemia prevalence of postpartum women in Mexico, and data and research on postnatal care in this country are very limited [8]. In Mexico 55% of the deliveries occur via Cesarean section (C-section), which, when improperly followed up, can lead to short- and long-term complications for the mother and baby [9, 10]. It is therefore imperative to document and monitor postnatal care data to identify and prevent any gaps in care during this period, which globally has the highest risk of mortality for both the mother and the baby [11]. Adequate nutrition is a critical component of healing and has been an important element of maternal care for Mexican women [12]. 

Mexico used to follow the *dieta de la milpa*, which provided enough macro- and micronutrients through meals based on corn, beans, squash, leafy greens, fruits, chilies, chocolate, and small amounts of protein [13]. However, for the last 30 years, this country has been undergoing a demographic, epidemiologic, and nutrition transition, including an increase in the non-communicable disease burden from high to low socioeconomic groups [14–16]. This transition has increased the levels of diseases related to malnutrition in all life stages, including pregnancy and postnatal periods [17]. Low-income women are a particular risk during the postnatal period, as socioeconomic status may influence food access, intake, and quality [18]. Cultural practices and dietary restrictions during the postnatal period are common around the world, including Mexico [19]. While there is a vast literature base on nutrition among postpartum women in Mexico, almost all is exclusive to breastfeeding practices and their impact or association on different outcomes for the mother and baby in Mexico [4, 5, 20–23]. To our knowledge, there is no recent literature that exclusively examines the dietary practices of Mexican mothers during the postpartum period to better understand the maternal diet of women in this country. The primary aim of the larger study documented the nutritional practices and knowledge that women in the Southern state of Oaxaca received and employed during their pregnancy and in the postpartum period [24] The present discussion builds upon these findings to elaborate on the nutritional postpartum practices of low-income women and their families in Southern Mexico.

## Methods

### Study design and conceptual framework

A qualitative study guided by the Ecological Systems Theory [25] and the Intersectionality Framework [26] was conducted among low-income women with at least one child under the age of five residing in the cities of Oaxaca City and Puerto Escondido, Oaxaca, Mexico, to gain a more detailed understanding of the nutritional practices of low-income postnatal women in Southern Mexico. The Ecological Systems Theory focuses on how human development is interconnected by multiple sociocultural and environmental layers [27], while the Intersectionality Framework helps understand the different experiences and social identities of one individual and how these intersect to shape privilege or oppression [28, 29]. Both frameworks were chosen to better understand the influence of the multiple relationship levels on the nutritional practices of women during the postnatal period, as well as the interaction and intersection of these with the multiple social positions each woman has after childbirth. This is important as a woman’s combined identities and the complex interplay of factors between her individual, relationship, and community converge to impact her nutritional practices during the postnatal period.

### Study setting

This study was conducted in the cities of Oaxaca and Puerto Escondido in southwestern Mexico. Both cities are located in the state of Oaxaca, which has some of the highest levels of poverty and food insecurity in the country, with over 25% of the population in this southern state living in extreme poverty [14, 30–32], 23% of the population currently experiencing moderate or severe food insecurity and close to 30% lacking access to nutritious and quality food [32]. Puerto Escondido is a coastal community located on the Pacific Coast and is in a tropical, high-humidity ecological zone whereas Oaxaca City is in a subtropical highland to semi-arid ecological zone [33]. Oaxaca is home to a rich agricultural base and culinary heritage shaped by both ecological and cultural diversity, Indigenous cultures, and a post-colonial history that make up this southern state [34–37]. 

### Study participants

The inclusion criteria comprised women aged 18 years or older who had at least one full-term pregnancy in the past five years and had lived in the state of Oaxaca full-time during the past five years. Participants in the city of Puerto Escondido, a smaller and more intimate community than Oaxaca City, were recruited through opportunistic sampling at a health center. While in the city of Oaxaca, the capital of the state, snowball and opportunistic sampling were used to recruit participants through word of mouth; in-person solicitation at health centers, hospitals, and markets; flyers posted at hospitals and health centers; and phone calls to patients from a health center. Participant recruitment lasted until thematic saturation was reached (*n* = 25).

### Data collection

Twenty-five semi-structured in-depth interviews were conducted between June and December 2023. The interviews were conducted in Spanish, the native language of the authors and participants, and lasted approximately between 30 and 45 min. The interviews in the city of Puerto Escondido (*n* = 16) were at the Lázaro Cárdenas Health Center, while in the city of Oaxaca, six interviews were conducted at the San Diego State University Oaxaca Center for Mesoamerican Studies and three at markets or local events, locations where participants were recruited. All interviews were conducted, audio-recorded, and transcribed by the primary investigator (MM).

Prior to the interview, participants completed a short socio-demographic survey as well as the 8-item adult version of the Latin American and Caribbean Scale of Food Security (ELCSA is the Spanish acronym) [38]. An interview guide, informed by investigators’ prior formative research along with the Ecological Systems Theory and the Intersectionality Framework, focused on the intersection of the individual, relationship, and community-level factors and the multiple social identities, particularly the physical health, of participants. The guide used broad open-ended questions to foster a discussion on women’s nutritional practices during pregnancy and the postpartum periods, including food acquisition, consumption of nutritional supplements, and nutritional education to guide the interviewing process. Key questions and example probes from the interview guide are found in Table 1.

**Table 1:** Key qualitative questions from the semi-structured interview guide on nutritional practices during the postnatal period among women in the cities of Oaxaca and Puerto Escondido, Mexico

Key question	Example probe
Related to food consumption and acquisition:	
1. What would you say were the foods you ate the most during the postpartum period after your last pregnancy?	Why did you consume these foods? How did you obtain these foods? For how long were these foods consumed? Were there any foods that you avoided to consume? If so, why?
2. For women who have delivered more than once, did you eat differently during the postnatal period after the different pregnancies?	If so, how do you eat differently? Why did you eat differently?
Related to nutrition education:	
3. Can you describe the nutritional education you received after delivery or during the postnatal period and from whom?	Who provided this information? What type of nutritional education did you receive from your health provider after delivery or during the postpartum period? If not from healthcare professionals, where or from whom would you say your nutrition knowledge that you used during the postnatal period comes from?
Related to nutritional supplements:	
4. For women who consumed nutritional supplements during the postnatal period: Can you tell me more about the supplements you consumed?	What type of supplements did you take? How did you obtain these supplements? How frequently would you say you consume these supplements? For how long did you consume these supplements? For the participants who stopped consuming nutritional supplements, could you provide me the reason why you stopped?

### Data analysis

All interviews were audio recorded and transcribed verbatim maintaining the original language for analysis to preserve participants’ meanings and nuance [39]. To tailor the interview guide and develop the codebook, the first author completed data collection and analysis concurrently. Two coders (MM and LC) independently read and coded all interviews using NVivo R1. A grounded theory approach was used for analysis as data was analyzed through emic coding concurrently with data collection [40–42]. The Ecological Systems Theory and the Intersectionality Framework were also used for the analysis of data and dissemination of results, as particular attention was given to the different individual (such as physical health, living location, age, and economic situation, to name a few) and relationship (such as family, social networks, and health professionals) levels that influenced the nutritional practices of the participants during the postnatal period. Coding consistency meetings were conducted between the coders to create new codes, discuss code definitions, and draw consensus on any discrepancies during the coding and analytic processes. Table 2 illustrates the initial codes and definitions that were identified during coding and analysis as they were refined to create the themes presented in this article.

**Table 2:** Theme generation and data analysis structure on nutritional practices during the postnatal period among women in the cities of Oaxaca and Puerto Escondido, Mexico (*n* = 25)

Overarching Theme	Codes	Code definition and description
Maternal diet after a C-section	Access to health care	Anything mentioned by participant related to having access to health care services during and after childbirth.
	Childbirth	Anything mentioned related to the childbirth process.
	Consumption of natural remedies	Anything related to consuming natural remedies such as plants, teas, infusions, for ailments related to postnatal care.
	Family	Anything related to her family during the postnatal period, such as financial/emotional support, sharing of knowledge.
	Food eaten during postnatal period	Any food mentioned by participant that was eaten during the postnatal period (after pregnancy and delivery).
	Foods in nutrition education	Any examples of foods mentioned related to what their health care provider (doctor, nurse, nutritionist, etc.) told them to eat after delivery.
	Woman’s health issues	Any health issue related to the mother during and after childbirth.
	Where knowledge comes from	Where the participant’s nutritional knowledge or information used during the postnatal period comes from (family, internet, friends, doctor, nutritionist, etc.).
Food practices during the breastfeeding period	Access to health care	Anything mentioned by participant related to having access to health care services during and after childbirth.
	Baby’s health/health issues	Anything related to the health or health issues related to the baby after birth.
	Breastfeeding	Anything related to breastfeeding.
	Consumption of natural remedies	Anything related to consuming natural remedies such as plants, teas, infusions, for ailments related to postnatal care.
	Family	Anything related to her family during the postnatal period, such as financial/emotional support, sharing of knowledge.
	Food eaten during postnatal period	Any food mentioned by participant that was eaten during the postnatal period (after pregnancy and delivery).
	Foods in nutrition education	Any examples of foods mentioned related to what their health care provider (doctor, nurse, nutritionist, etc.) told them to eat after delivery.
	Where knowledge comes from	Where the participant’s nutritional knowledge/information used during the postnatal period comes from (family, internet, friends, doctor, nutritionist, etc.).
Nutrition supplements after the delivery	Access to health care	Anything mentioned by participant related to having access to health care services during and after childbirth.
	Economy	Anything mentioned by participant related to the economy, money, or economic problems during the postnatal period.
	Frequency of consumption of nutritional supplements	Anything related to how frequently nutritional supplements were consumed during the postnatal period.
	How were nutritional supplements obtained	Anything mentioned by participant related to how nutritional supplements were obtained during the postnatal period
	Reasons for not taking supplements	Anything related to not using or stopping the consumption of supplements during the postnatal period.

### Ethical considerations

This study was granted ethical approval by San Diego State University Institutional Review Board (Record Number HS-2023-0108). All women in this study provided written informed consent prior to participation. All identifiable participant information was anonymized, securely coded, and stored.

## Results

### Sample characteristics

The majority of participants were between the ages of 20 and 30 (52%, range: 20–42). Most were born in the state of Oaxaca (92%), were currently living with a partner or married (88%), had less than a high school educational attainment (76%), were stay-at-home mothers (68%), considered themselves of indigenous belonging (56%), reported having had an average of one to three pregnancies (92%; range: 1–7), and were currently low food insecure (60%).

### Emergent themes

We identified three emergent themes driven by the Intersectionality Framework and the Ecological Systems Theory to understand women’s multiple individual characteristics and social and ecological factors that influence women’s nutrition decisions and practices during the postnatal period: (1) maternal diet after a C-section; (2) food practices during the breastfeeding period; and (3) nutrition supplements after the delivery. These themes were linked particularly to the individual-level characteristics and the social identities of the participants after childbirth as well as the relationship and community-level support for the nutritional practices during the postnatal period. Each theme is described in detail below.

#### Theme 1. Maternal diet after a C-section

The most common theme in postpartum nutritional practices among participants was the diet followed by women who gave birth to their child via C-section, compared to a few participants who delivered vaginally and specified that they were not given specialized nutritional instructions or diets. Some participants who delivered via C-section remembered having what they considered very restrictive diets after childbirth.

*“Eating salads*,* vegetables*,* and meat—but without salt or sugar—I wasn’t eating anything at all.”* (Participant #23, age 41, Puerto Escondido).

According to participants who had a C-section, the most common foods recommended to eat during the recovery period included vegetables, fruits, soups (such as vegetable or chicken soup), rice, eggs, and cheese. Related to what to drink during this period, water is mentioned as the beverage to drink. A participant mentioned that sodas and other forms of carbonated drinks were prohibited during this period.

*“(doctors told me to eat) what are broths*,* vegetables*,* but without tortillas and without bread”* (Participant #15, age 38, Puerto Escondido).

*“Just broth*,* just vegetables*,* just fruit. No fat at all. Well*,* that was because of the C-section.”* (Participant #18, age 22, Puerto Escondido).

Consumption of pork, seafood, or foods with a high content of fat were the foods more frequently mentioned by participants as being restricted from their diets after the C-section. Bread, tortillas, and spicy foods were also prohibited for a few participants. Participants also mentioned that to prevent high content of fat or the use of oil, some foods such as eggs, cheese, or meat could only be eaten if they were grilled or boiled and not cooked in some other form.

*“Because of the C-section*,* pork is bad for you”* (Participant #6, age 24, Oaxaca City).

*“It was pure nourishment—but completely natural; there was no oil involved*,* because I had a C-section.”* (Participant #10, age 20, Puerto Escondido).

The time that these diets were followed by women after their C-section delivery varied from one week to one year; however, two months was the most frequent period mentioned among participants.

*“For nearly two and a half months*,* I was careful not to eat all those things—because*,* supposedly*,* it was bad for my C-section recovery. And yes*,* I really did watch what I ate for two months. I continued to be careful for a little while longer—specifically regarding those foods—for two and a half months in total; and at that point*,* I started eating the same things I used to eat before.”* (Participant #20, age 21, Puerto Escondido).

Some participants mentioned these dietary practices were recommended by health professionals right after the delivery of the child. However, others mentioned that the food recommended and the amount of time to follow these practices were taught by older family members, such as mothers, grandmothers, and mothers-in-law. Some of the participants mentioned that a specific diet, particularly avoiding seafood or pork, was to prevent constipation or to prevent the C-section wound from opening or getting infected.

*“Parents get you used to other things*,* like you can’t eat pork*,* or you can’t eat fish because it will infect your (C-section) wound*,* so they stop giving us certain foods that we don’t consume during that two-month period.”* (Participant #23, age 41, Puerto Escondido).

*“She (grandmother) told me (I) couldn’t eat fish because something happened to a colleague of hers—it (wound) opened up a little because she ate shrimp.“* (Participant #10, age 20, Puerto Escondido).

#### Theme 2. Food practices during the breastfeeding period

Another common theme among participants was the food practices that postpartum women followed while breastfeeding. Similar to some participants who delivered via C-section, a few women mentioned having restrictive diets during their postpartum period because they were breastfeeding their child.

*“Yes*,* I changed my diet a lot. The first month was quite strict at home because we (participant and her natal family) believed that you only take care of yourself for a month. So*,* I followed a very strict diet because of breastfeeding.”* (Participant #18, age 34, Puerto Escondido).

*Atole*, a traditional hot beverage made from ground corn dough, was commonly mentioned by some participants as a food they consumed during the postpartum period to increase the milk supply for breastfeeding. This was to be consumed up to a few times a week and was suggested by healthcare professionals, such as nurses, after the delivery or during follow-up appointments, as well as by participants’ mothers and mothers-in-law.

*“Atole*,* to help the milk come in. To help the milk come in and have enough for the baby to drink.”* (Participant #12, age 31, Puerto Escondido).

Besides consuming *atole*, a few women who gave birth vaginally described having the same diet as what they ate before becoming pregnant while breastfeeding; however, some did have a more restrictive diet while breastfeeding that included broths, vegetables, fruits, and juices. A few women also mentioned some foods that were not recommended during the postpartum period while breastfeeding, such as beans and corn, as they would lead to minor health issues for the baby.

*“(I eat) the same (food). Fish*,* same thing. I mean*,* beef. Whatever.”* (Participant #14, age 34, Puerto Escondido).

*“They say not to eat certain foods*,* like beans*,* because that’s what gives the girl gas. That’s what they say. And in fact*,* well*,* my mother-in-law has also told me that corn tamales also cause gas; she says that’s what triggers it too.”* (Participant #16, age 22, Puerto Escondido).

*“It (diet) was very paltry. At home*,* my mom only gave me broths*,* soups*,* egg broth*,* chicken soup*,* chicken with vegetables*,* and more broths and more broths. And no tortillas*,* just toast. My delivery was natural*,* but my mom still took great care of me for about 40 days.”* (Participant #18, age 34, Puerto Escondido).

While many participants noted that they consumed *atole* for up to three months, most indicated that they breastfed for six months, at which time that they started introducing mashed vegetables to their child’s diet.

*“I consumed it (atole) for about two or three months*,* I think. I gradually stopped taking it*,* but I did take it. That’s why they told me to drink a lot so my (breast) milk would come in*,* and then to take care of myself*,* to avoid the cold*,* and to bathe at a reasonable hour so the (breast) milk wouldn’t get cold and harm the baby.”* (Participant #12, age 31, Puerto Escondido).

#### Theme 3. Nutrition supplements after the delivery

Consumption of postpartum nutrition supplements in the form of vitamins, folic acid, and iron in tablets or injections was mentioned by participants. These were always suggested by health care professionals. The reason why some of the participants continued consuming supplements included replacing blood lost during delivery and replenishing nutrients depleted during pregnancy.

“*The thing is*,* I lost a lot of blood (during delivery)*.” (Participant #8, age 42, Oaxaca City).

*“Throughout my entire pregnancy—before and even afterwards—they kept sending me for (iron and folic acid) because I had lost so much weight.”* (Participant #18, age 34, Puerto Escondido).

For participants who used supplements during the postpartum period, the length of time these supplements were used varied by participants from a month to up to six months, with three months being the most frequent time provided by participants. One participant was still taking iron supplements at the time of the interview. Reasons for stopping consuming supplements during the postpartum period included no longer needing to consume supplements, already consuming enough supplements, and economic issues where the participant was no longer able to afford buying supplements.

*“Consequently*,* I only took those medications for three months*,* even though I still needed another three months’ supply; this was because his father also underwent surgery shortly thereafter—creating yet another expense—on top of the costs for his food*,* my food*,* the baby’s food*,* and all the baby’s other needs. So*,* that is why I tell you: there were indeed times when we found ourselves facing such hardship.”* (Participant #23, age 41, Puerto Escondido).

## Discussion

This qualitative study explored the nutritional practices of women during the postnatal period in two cities of the southwestern state of Oaxaca, Mexico, one on the coast and the other in a major urban space in the valley. Our results highlight the lived experience of 25 women in the cities of Puerto Escondido and Oaxaca City. Our findings suggest that the nutritional practices during the postnatal period are driven by three health outcomes of the perinatal period: delivering via a C-section, practicing breastfeeding, and requiring additional nutrients after delivery.

Our results found that women in Oaxaca City and Puerto Escondido who deliver via a C-section have a more restrictive diet during the postpartum period than women who deliver vaginally; however, a variety of milpa-based traditional foods, such as vegetables and fruits, are the main source of nourishment for these women during the postnatal period. As dietary patterns are associated with sociodemographic characteristics, such as socioeconomic status and area of residence [43, 44], our findings align with results from the National Health and Nutrition Surveys that found that women and people of lower socioeconomic status, mainly from Southern Mexico, have a higher quality of diet in terms of its nutritional value [43]. As Oaxaca is one of the states with higher levels of food insecurity in the country [32], future research could evaluate the coping mechanisms low-income women in this state use to have a high quality of food during the postnatal period.

Also related to the restrictive diets for postnatal women who delivered via C-section were the foods that were avoided as a result of a belief that they impact C-section stitches opening or getting infected, such as seafood, pork, foods with a high fat content, and spicy food. In a study on food taboos in Guadalajara, Mexico, chili was one of the most common foods avoided after childbirth; however, this was related to breastfeeding practices and not delivering via C-section [45]. While the practice of avoiding fish and oil was also found in other studies done in a semi-urban community from the central state of México (Estado de México), and a rural area of the central state of Hidalgo, respectively, the reason for this was different, as it was specific for pregnant and breastfeeding women in the first study, and it was not specified for a time period among women in the second study [23, 46]. However, the practice of avoiding these foods to prevent a C-section infection has been found in postpartum women in other countries such as India, Bangladesh, and several countries in Africa [47–49]. 

Breastfeeding women in Puerto Escondido and Oaxaca City also described restrictive diets during the postnatal period, which match results from other studies done in different regions of Mexico. For example, similar to our results, studies in rural areas of Hidalgo and Estado de Mexico also mentioned *atole* as something to drink to increase the milk supply among women who breastfeed, as well as restricting some foods and vegetables during this period [23, 46]. However, some of the restrictions in food that our participants mentioned are not practiced in other regions. For example, a study in Guadalajara, Jalisco, one of the biggest cities in the country, found that corn-based foods, particularly tortillas, were the most consumed items during the breastfeeding period [50], while some of our participants stayed away from foods made of corn, such as *tamales* (which are typically made with pork fat), to prevent health issues in their babies; however, it is important to state that *atole*, a main gruel type of food consumed by participants when breastfeeding, is a corn-based beverage and thus challenges the restriction on corn-based foods. This may be an area to investigate further. Many of our participants stated consuming broths and juices as their main diet during breastfeeding; it is possible that our participants focused on consuming corn as part of a “hot” drink, while excluding consuming corn in a solid form that could be cooked with foods, such as pork fat. Given that the breastfeeding rates across Mexico are lower than the recommendation from the WHO, particularly after the first 6 months [51], it is imperative to further understand the reasons why women decided to breastfeed or not breastfeed their infant, including the nutritional practices around this activity to best help mothers continue this practice for the recommended time.

Several participants followed a strict diet, either because of the C-section or because they were breastfeeding, for 30 to 60 days after the delivery. This is typical of *“ la cuarentena”* (also known in English as postpartum confinement or quarantine). The *cuarentena* is the immediate period after delivery that lasts for 40 days, traditionally common in Mexico, that involves a period of rest for the mother and where household support (such as cooking and cleaning) is done mainly by female family relatives [52, 53]. In our study, these customs continued to be practiced among our participants nutrition-wise, and these practices came mainly from participants’ mothers and mothers-in-law, as they were the primary sources of nutritional information and at times were in charge of the participant’s diet during the postnatal period, even when the term *cuarentena* was not adhered to as frequently. Describing nutritional customs during the postnatal period without including the term *cuarentena* or excluding nutrition customs when discussing the *cuarentena* is not uncommon in recent Mexican literature. A study in the southeastern state of Veracruz on *cuarentena* showed that women continue to observe these culturally prescribed practices; however, no information was provided on the nutritional practices that were followed during the *cuarentena* [54]. While a study on puerperium that briefly used the term *cuarentena* interchangeably found that close to 65% of Purépecha Indigenous women in the state of Michoacan continue to follow nutritional customs and practices during puerperium, even though they follow fewer customs for physical and general care [55]. Since cultural nutritional practices continued to be followed in urban and rural Mexico during the postnatal period, it is crucial to further understand what the *cuarentena* currently stands for as well as the different traditional practices, particularly nutrition-wise, for integrating a culturally sensitive approach to health care policies at the local, state, and national level.

Related to the use of nutritional supplements during the postpartum period, the WHO recommends postpartum oral iron and folate supplementation for 6 to 12 weeks for some women with an increased risk of anemia [56]. The only participants in our study who used nutritional supplements, mainly iron and folic acid, during the postnatal period were the ones who were prescribed them by a professional health provider to replenish depleted nutrients and for recovery after delivery. These results argue that postnatal women in Oaxaca and Puerto Escondido only use nutritional supplements when necessary. However, while not common among participants, it was mentioned by a few participants in our study that postnatal supplement consumption was stopped due to financial issues. There is limited literature on postnatal care in Mexico; however, another study in Oaxaca and five other Mexican states found that maternal nutritional care compliance during the postpartum period is very low (from 5.7% to 17%) [12]. Postpartum care is frequently underemphasized compared to antenatal care around the world, including Mexico. Recent research found that as high as 30% of women do not have postnatal care visits within 6 weeks after their discharge from delivering the baby [8, 57]. These existing data, along with our findings, suggest more research on maternal adherence to nutrition supplements in Mexico, particularly iron and folic acid, are needed to better understand the prevalence of supplement consumption or lack thereof.

While most of the information provided by participants was on the foods they consumed during their postnatal period, several provided the source of where the information from those practices came from. While the nutrition supplements were exclusively recommended by health professionals, the nutritional practices information from women recovering from C-sections, as well as for breastfeeding women, came from family members, more commonly mothers, grandmothers, and mothers-in-law. While there are qualitative studies in Mexico that describe the role of mothers, sisters, and grandmothers in postnatal women’s nutrition practices, particularly while breastfeeding [58, 59], quantitative studies should also be completed around the country to better understand the knowledge and practices these women share with postnatal women and learn if there are any gaps in nutrition that are left from what family members know and what healthcare providers do and do not provide after delivery.

### Strengths and limitations

This study provides rich information regarding nutritional practices and lived experiences of women during the postpartum period. This is one of the few studies that examines the nutritional practices of postpartum women [1], and to our knowledge, the first one that uses the Ecological Systems Theory and the Intersectionality Framework in postnatal nutrition in Mexico. This study is not without limitations. Given the small sample size (*n* = 9) in the city of Oaxaca, this study may not capture the complete range of different dietary behaviors practiced by women during the postnatal period in the capital of the state of Oaxaca; however, by using qualitative methods for this study, our goal was not to achieve population generalizability. Also, this study focused on women from a lower socioeconomic level in urban areas. Future work that focuses on more diverse populations would help to elucidate differences in nutritional practices and patterns by sociodemographic characteristics, such as socioeconomic status and geographical location. Another possible limitation is potential recall bias, as we interviewed women with children up to the age of five, and for some women, their postpartum experiences had already passed for several years.

## Conclusions

This study highlights the food practices among women in Puerto Escondido and Oaxaca during the postnatal period, which are driven by customs and focus on the recovery of the mother and the development of the infant. Still, restrictions in women’s food choices could impact the quality of diet as well as outcomes for both mother and baby, so future research is required to identify any potential gaps in nutrition for these women during puerperium. Until recently, Mexico did not have an official feeding guide for pregnant and lactating women [60]. In 2025 the Mexican government published the Healthy and Sustainable Dietary Guidelines for the Mexican Population 2025–2030, which included specific guidelines for pregnant and lactating women [61]. Our study adds culturally relevant information for future versions of this guide, particularly for a future regional guideline of the state of Oaxaca. Given the limited data of postnatal care in this country, our research provides new evidence on the influence of individual, group, and structural-level factors and their interaction with the multiple social positions and characteristics of women in their nutrition practices during the postnatal period. Still, more research is required to further understand and identify barriers and enablers to nutritional practices during puerperium in order to develop targeted interventions, policy changes, and any broader initiatives that focus on the health and nutrition of postnatal women in the state of Oaxaca.

## Data Availability

Excerpts of the data are in this publication. Participants’ transcripts are not available to share as they contain confidential information. For more information, please reach the corresponding author.

## Abbreviations

C-section: Cesarean section
WHO: World Health Organization
